# Two Key Substitutions in the Chromophore Environment of mKate2 Produce an Enhanced FusionRed-like Red Fluorescent Protein

**DOI:** 10.32607/actanaturae.27545

**Published:** 2025

**Authors:** D. A. Ruchkin, A. S. Gavrikov, D. V. Kolesov, A. Yu. Gorokhovatsky, T. V. Chepurnykh, A. S. Mishin, E. G. Maksimov, N. V. Pletneva, V. Z. Pletnev, A. M. Pavlova, V. A. Nikitin, A. M. Bogdanov

**Affiliations:** Shemyakin–Ovchinnikov Institute of Bioorganic Chemistry, Moscow, 117997 Russia; Faculty of Biology, M.V. Lomonosov Moscow State University, Moscow,119992 Russia; Pirogov Russian National Research Medical University, Moscow, 117997 Russia; Department of Photonics, İzmir Institute of Technology, İzmir, 35430 Turkey

**Keywords:** RFP, FusionRed, mKate2, fluorescent protein, fluorescence lifetime

## Abstract

Red fluorescent proteins (RFPs) are often probes of choice for living tissue microscopy and whole-body imaging. When choosing a specific RFP variant, the priority may be given to the fluorescence brightness, maturation rate, monomericity, excitation/emission wavelengths, and low toxicity, which are rarely combined in an optimal way in a single protein. If additional requirements such as prolonged fluorescence lifetime and/or blinking ability are applied, the available repertoire of probes could dramatically narrow. Since the entire diversity of conventional single-component RFPs belongs to just a few phylogenetic lines (DsRed-, eqFP578- and eqFP611-derived being the major ones), it is not unexpected that their advantageous properties are split between close homologs. In such cases, a systematic mutagenetic analysis focusing on variant-specific amino acid residues can shed light on the origins of the distinctness between related RFPs and may aid in consolidating their strengths in new RFP variants. For instance, the protein FusionRed, despite being efficient in fluorescence labeling thanks to its good monomericity and low cytotoxicity, has undergone considerable loss in fluorescence brightness/lifetime compared to the parental mKate2. In this contribution, we describe a fast-maturing monomeric RFP designed semi-rationally based on the mKate2 and FusionRed templates that outperforms both its parents in terms of molecular brightness, has extended fluorescence lifetime, and displays a spontaneous blinking pattern that is promising for nanoscopy use.

## INTRODUCTION

Current bioimaging techniques recruit a vast diversity of fluorescent probes; among those, genetically encoded fluorophores such as fluorescent proteins (FPs) are in favor. FPs enable highly specific intracellular labeling, live-cell super-resolution, fluorescence lifetime imaging microscopy (FLIM), etc. [1, 2]. In turn, red fluorescent proteins (RFPs), a polyphyletic group [3, 4, 5, 6] of anthozoan FPs emitting in the red region of the spectrum, are of particular relevance in whole-body and/or deep-tissue imaging, owing to their enhanced detectability within the optical transparency window characterizing the local absorption minimum of animal tissues at wavelengths of ~ 600–1200 nm [7, 8, 9].

Among existing RFP variants, FusionRed [10] is often the probe of choice for live-cell imaging (including the visualization of fine subcellular structures) thanks to its "supermonomericity," i.e., an ability to maintain a highly monomeric state even at the high local concentrations that are typical of specialized localizations within mammalian cells [11, 12], as well as its low acid sensitivity and toxicity. It is thus supposed to be used as a probe fused to the proteins of interest without affecting their natural activities and spatial structures, or can function as a fluorescent core of genetically encoded indicators [13, 14, 15, 16]. Along with this, there are definite drawbacks that take from the practical value of FusionRed as a multipurpose fluorescence tag and call for further improvement of this RFP. In that perspective, the issue of the modest molecular brightness of FusionRed has been addressed in the elegant work by Jimenez lab, where both directed evolution [17] and semi-rational design [18] were utilized to engineer brighter variants of FusionRed (specifically, FusionRed-MQV [19] has an ~ fourfold higher molecular brightness over the parental RFP, although its emission peak comes with a 20-nm hypsochromic shift). The high-resolution spatial structure of FusionRed revealed that almost half of its molecules carry an immature chromophore [20]; this feature impinges on the effective brightness (well under the level expected based on the measured molecular brightness) of FusionRed as a fluorescence probe, suggesting further room for improvement via a structure-based design of daughter RFP variants.

Importantly, FusionRed is a descendant of the mKate2 protein [21] that emits at 633 nm (its emission maximum is 25-nm red-shifted relative to that of FusionRed) and is currently the brightest monomeric far-red FP. FusionRed differs from mKate2 in 17 amino acid substitutions introduced semi-rationally through several consecutive rounds of mutagenesis [10]: Hence, there is no consolidated picture that describes the particular role of every substitution, specifically since the structural foundations of the spectral differences (including the extinction coefficient, fluorescence quantum yield and lifetime, as well as the excitation/emission maxima positions) between the FusionRed and mKate2 proteins are not clear enough. Based on an analysis of the spatial structure of FusionRed [20], one can assign an essential role to three residues in the chromophore environment: Arg/Lys-67, Cys/Ala-158, and His/Arg-197 (FusionRed/mKate2, respectively). Here, we systematically studied the influence of these residues on the properties of both proteins through exhaustive reciprocal site-directed mutagenesis. Among the representatives of the library obtained, there is one that is remarkable: mKate2-K67R/R197H, which shows a striking similarity in its steady-state absorption and fluorescence spectra to those of FusionRed and is 2.2-fold brighter than the latter. This RFP inherits the advantages of both sister proteins; namely, it demonstrates a monophasic fluorescence decay similarly to mKate2 and performs well as a fusion tag like FusionRed. Interestingly, purified mKate2-K67R/ R197H possesses a well-marked pattern of spontaneous fluorescence blinking that might be promising for use in super-resolution microscopy.

## MATERIALS AND METHODS

**Site-directed mutagenesis**

A modified IVA-cloning [22] procedure was applied to produce the site-specific mutants of mKate2 and FusionRed. The genes of the chosen RFPs, cloned into the pQE-30 vector backbone (Qiagen, Germantown, Maryland, USA) using BamHI/HindIII endonuclease sites, were utilized as primary templates. The forward oligonucleotides were designed to have a 5’-terminal 15– to 20-nt length region homologous to the template DNA (needed to provide bacterial recombination), followed by a triplet with the mutation of interest and a 3’-terminal priming region designed to anneal at 60°C. The reverse oligonucleotides consisted of a similar recombination-guide part of 15–20 nt and a 3’-terminal priming sequence; both made up an annealing temperature of 60°C when possible. In cases of higher calculated annealing temperatures, the 5’-end fragment was considered partially annealing. The 3’ and 5’ terminal bases of both primers were selected in such a way as not to pair complementarily in order to avoid self-annealing of long oligonucleotides when possible; simultaneously, the terminal 3’-nucleotides of both primers were selected, by design, to form a strong complementary pair with the template sequence. The reverse primer could never anneal to the forward one with 3’-terminus resulting in a blunt-end. PCR was performed using the standard Phusion Polymerase (ThermoFisher, Waltham, Massachusetts, USA) protocol and lasted 35 cycles; the template DNA made up for a total of 50 ng per reaction. The primers used had the following sequences:

(a) FusionRed-R67K:

Forward – 5’-agcttcatgtacggcagcaaaaccttcatcaagcaccctccgg-3’

Reverse – 5’-gctgccgtacatgaagctggtag-3’

(b) FusionRed-C158A:

The mutant was engineered in the previous study [20].

Forward – 5’-cggcggcctggaaggcgcagcagacatggccctgaagctcg-3’

Reverse – 5’-tgcgccttccaggccgccgtcagcggggtacatcgtctcg-3’

(c) FusionRed-H197R:

Forward – 5’-ggcgtctacaacgtggacagaagactggaaagaatcaaggaggc-3’

Reverse – 5’-gtccacgttgtagacgccgggcatcttgaggttcgtagcg-3’

(d) mKate2-K67R:

Forward – 5’-agcttcatgtacggcagcagaaccttcatcaaccacacccaggg-3’

Reverse – 5’-tgctgccgtacatgaagctggtag-3’

(e) mKate2-A158C:

The mutant was engineered in the previous study [21].

Forward – 5’-ggcctggaaggcagatgcgacatggccctgaagctcg-3’

Reverse – 5’-tctgccttccaggccgccgtcagcggggtacag-3’

(f) mKate2-R197H:

Forward – 5’-ggcgtctactatgtggaccacagactggaaagaatcaaggaggc-3’

Reverse – 5’-gtccacatagtagacgccgggcatcttgaggttcttagcg-3’

**Protein expression and purification**

The FP variants were expressed in the E. coli XL1- Blue strain for 72 h at 37°C. After centrifugation, bacterial biomass was resuspended in PBS (GIBCO, ThermoFisher Scientific, Waltham, Massachusetts, USA), pH 7.4, and treated with ultrasound using a Sonics Dismembrator (Fisher Scientific, Pittsburgh, Pennsylvania, USA). The proteins were then purified using the TALON metal-affinity resin (Clontech, Mountain View, California, USA) added earlier and washed in PBS according to the manufacturer’s protocol, solubilized using 0.3 mM imidazole (pH 8.0). The protein eluates were then desalted and concentrated by ultrafiltration with Amicon Ultra 0.5 10K columns (Merck Millipore, Burlington, Massachusetts, USA). The resulting concentrated protein solution (typically ~5 mg/mL) was ready for use in a SDS-PAGE analysis or spectroscopy or could be stored for a short time at 4°C until use.

**Steady-state absorption and fluorescence spectroscopy**

The absorbance and fluorescence spectra were recorded using a Cary100 UV/VIS spectrophotometer and a Cary Eclipse fluorescence spectrophotometer (Agilent Technologies, Santa Clara, California, USA), respectively. A protein solution in PBS (pH 7.4) was used in all the cases. The fluorescence quantum yields and extinction coefficients were determined as described earlier [20].

**Monomericity testing**

Gel filtration. Gel filtration experiments were performed using a Superdex® 200 Increase 10/300 GL column (Cytiva, Uppsala, Sweden) equilibrated with a 20 mM sodium phosphate buffer (pH 7.4) containing 150 mM NaCl at 24°C at a flow rate of 0.75 mL/min. The column was connected to an Agilent 1260 Bio- Inert LC system equipped with an in-line Agilent 1260 diode array detector and an Agilent 1260 fluorescence detector and calibrated using cytochrome C (12.4 kDa), carbonic anhydrase (29 kDa), bovine serum albumin (66 kDa), alcohol dehydrogenase (150 kDa), b-amilase (200 kDa), and ferritin (450 kDa). The calibration details are presented in Fig. S6 and Table S1. The equipment was controlled by the Agilent OpenLAB CDS ChemStation Edition C.01.07 SR3 software.

OSER assay. The OSER assay was undertaken in two variants. The first one, based on HeLa cells, was similar to that described in ref. [12]. The cells were transfected with the FuGENE® HD Transfection Reagent (Promega, Woods Hollow Road, Madison, USA) according to the commercial protocol. Images were acquired with wide-field fluorescence microscopy using a modified Leica 6000LX inverted microscope equipped with an mCherry filter cube (see the Widefield fluorescence microscopy section). The images were processed using Fiji ImageJ distribution (version 2.9.0/1.54b). Whorl-like structures were then identified according to the guidelines elaborated by Constantini et al. [23]. Due to the lack of whorl-like structures in more than 80% of the transfected HeLa cells and the resulting considerable difficulty in capturing enough whorl-possessing HeLa cells for a valid statistical analysis, the ratios between the mean fluorescent intensities of the nuclear envelope and whorl-like structures were not calculated.

The second variant of the OSER assay was similar to that described in ref. [23]. U2OS cells were transfected with polyethyleneamine (PEI, Sigma-Aldrich, Saint Louis, Missouri, USA). The observation was performed 18 h post-transfection; the images were analyzed using the same Fiji ImageJ software to extract the mean NE and OSER signals. Not less than three linear ROIs for NE were traced for each cell using the "straight" tracing instrument; the "freehand" instrument was used for OSER ROIs. The ratios were calculated using the GraphPad Prism10 software.

Engineering of mammalian constructs. Mammalian expression plasmids encoding fusions of Diogenes with vimentin (vimentin-Diogenes), lifeact (life act-Diogenes), ensconsin (ensconsin-Diogenes) and cytokeratin (Diogenes-cytokeratin), as well as the fusion with the cytoplasmic end of an endoplasmic reticulum signal anchor membrane protein (CytERM; used in the OSER assay), were assembled using Golden Gate cloning according to the MoClo standard procedure [24, 25, 26]. Each transcriptional unit for mammalian expression included a CMV promoter, a coding sequence for the fusion protein, and the SV40 terminator. All Golden Gate cloning reactions were performed in the T4 ligase buffer (SibEnzyme, Moscow, Russia) with 10 U of T4 ligase, 20 U of either the BsaI or BpiI restriction endonuclease (ThermoFisher, Waltham, Massachusetts, USA), and 100–200 ng of the DNA of each fragment. The assembly reactions were performed under the following conditions: 30 cycles of 37°C and 16°C incubation (90 s at 37°C, 180 s at 16°C).

**Widefield fluorescence microscopy**

Widefield fluorescence microscopy was performed with a Leica 6000LX inverted microscope equipped with a Leica HCX PL APO 100X/1.40–0.70NA oil immersion objective, a Zyla sCMOS camera (Andor, Oxford, UK), and a CoolLED pE-300 light source. An mCherry cube filter set (Leica, Wetzlar, Germany) was used (excitation filter: 560/40, emission filter: 630/75). The typical illumination power ranged from 1 to 5 W/cm2 with exposure times ranging from 50 to 150 ms.

**pH-stability measurement**

A set of pre-made buffer solutions with pH ranging from 3 to 10.55 was used to prepare the protein samples; the solutions contained 130 mM KCl, 30 mM NaCl, 0.5 mM MgCl_2_, 0.2 mM EGTA, and 30 mM HCl– NaH2C6H5O7 (pH 3.0–4.5) or 15 mM KH_2_PO_4_–Na_2_HPO_4_ (pH 5.0–7.5) or 20 mM Na_2_B_4_O_7_–HCl/NaOH (pH 8.0– 11.0) [27]. Each probe contained 5 μg/mL of the purified and desalted RFP. For each sample, the emission spectra were measured with a Cary Eclipse Fluorescence Spectrometer twice for each of three temporary points (immediately after preparation, after 3 min and 5 min), with a total of six measurements per sample in a spectral range from 560 nm to 700 nm at λex = 540 nm using a 5 nm ex/em slit, an equal photomultiplier (PMT) voltage, and the scanning speed values. The fluorescence intensity values at the emission maxima were averaged from six reads. The averaged data from all pH points for each RFP were normalized to the maximum value within the set and plotted on a graph with standard deviations. The sigmoidal regions of the graphs were fitted (4PL logistic curve, 95% confidence, n = 6) in GraphPad Prism10; the pKa of each protein was defined at a read of 0.5 on the fitting curve.

**Fluorescence lifetime measurements**

Nanosecond and picosecond setups. Measurements were made using a time-resolved (TCSPC) miniTau fluorescence spectrometer (Edinburgh Instruments, Livingston, UK) in a 20 ns window divided into 2,048 time channels. The fluorescence was excited using: (i) an EPL-450 picosecond laser (Edinburgh Instruments, Livingston, UK) with a central emission wavelength of 445.6 nm, a pulse width (FWHM) of ~ 90 ps@10 MHz driven at a repetition rate of 20 MHz; (ii) an EPLED-590 nanosecond pulsed LED (Edinburgh Instruments, Livingston, UK) with a central emission wavelength of 590 nm and a pulse width (FWHM) of ~ 1.3 ns driven at a repetition rate of 20 MHz. The photons were counted in a spectral range of 575–625 nm. The data processing, visualization, and determination of χ^2^ (Pearson’s test) were performed using the Fluoracle 2.5.1 software (Edinburgh Instruments, Livingston, UK).

Femtosecond setup. The fluorescence decay kinetics of RFPs were recorded by a single-photon counting (SPC) detector with an ultra-low dark count rate (HPM-100-07C, Becker & Hickl, Germany) in the 620/10 spectral window and adjusted by an ML-44 monochromator (Solar, Belarus). Fluorescence was excited at 590 nm (repetition rate, 80 MHz; pulse width, 150 fs; optical power, 5 mW) using the second harmonics (ASG-O, Avesta Project LTD, Moscow, Russia) of a femtosecond optical parametric oscillator (TOPOL-1050-C, Avesta Project LTD.) pumped by a Yb femtosecond laser (TEMA-150, Avesta Project LTD). The emission signal was collected perpendicular to the excitation beam. The sample temperature was stabilized during the experiment at 25°C with a cuvette holder (Qpod 2e) with a magnetic stirrer (Quantum Northwest, USA). The SPCM Data Acquisition Software v. 9.89 (Becker & Hickl, Germany) was used for data acquisition. The SPCImage software (Becker & Hickl, Germany) was used for the exponential fitting of fluorescence decays considering the incomplete decay of RFPs due to the high repetition rate. Post-processing and visualization of the collected data were performed using the Origin Pro 2018 software (OriginLab Corporation, USA).

**Photostability measurements**

Purified proteins, low excitation intensity. For the photobleaching experiments, the RFPs immobilized on TALON metal-affinity resin beads were imaged. Measurements were performed using a DMIRE2 TCS SP2 laser scanning confocal inverted microscope  (Leica Microsystems, Wetzlar, Germany) equipped with an HCX PL APO lbd.BL 63× 1.4NA oil objective and a 1.2 mW HeNe laser. The red fluorescent signal was acquired using the 543 nm excitation laser line and detected within a 560–670 nm spectral range. The selected field of view (16× zoom) was scanned in the time-lapse (between frames) mode, wherein the sequence of detection and bleaching frames was repeated 500–1,500 times without delay. To detect the red fluorescence signal, a 10%–20% laser power and PMT voltage of 700–800 V were used. For fluorophore photobleaching, the 100 % laser power (yielding about 2 W/cm2 power density) was used. The fluorescence data were all background-subtracted, averaged (n = 5), and normalized to the maximum value. A LaserCheck (Coherent, Saxonburg, Pennsylvania, USA) power meter was used to measure the total power of the excitation light after the microscope’s objective. The light power density (W/cm2) was estimated by dividing the total power by the area of the laser-scanned region.

In cellulo measurements, high excitation intensity. For the photobleaching experiments, the fluorescence signal of the RFPs, transiently expressed in the HeLa cell culture and lacking a specific intracellular targeting signal, was acquired. Measurements were performed using a Nanoimager S (ONI, Oxford, UK) microscope equipped with an Olympus UPlanSApo ×100 NA 1.40 oil immersion objective, a 561 nm laser, a 560 nm on-camera beam splitter, and a Scope8 sCMOS camera. The cells were irradiated in the epifluorescence mode with the 561 nm laser at a power density of 800 W/cm2 with simultaneous continuous signal recording and minimal delays between frames. Data analysis was performed using the FiJi ImageJ 1.53f51 software [28].

**Single-molecule localization microscopy**

Super-resolution BALM imaging of the cytoskeleton of cultured mammalian cells was performed as follows. Immediately before imaging, the cell medium was replaced with the minimal essential medium (MEM, Sigma-Aldrich, Saint Louis, Missouri, USA) supplemented with 20 mM HEPES. Single-molecule localization super-resolution imaging of living cells was performed using a Nanoimager S (ONI, Oxford, UK) microscope equipped with an Olympus UPlanSApo ×100 NA 1.40 oil immersion objective, a 561 nm laser, a 560 nm on-camera beam splitter, and a Scope8 sCMOS camera. Imaging was performed using the following imaging condition set: a 2 kW/cm2 561 nm laser and 16.7 ms frame time (60 fps acquisition speed). The imaging procedure with additional photostimulation by 405-nm laser flashes had the following conditions: imaging by a 561 nm laser operating at 2 kW/cm² was accompanied by 405 nm laser flashes with a duration of 0.4 s and an illumination density of ~ 215 W/cm², applied every 22 s. The frame recording time was 16.7 ms, and the acquisition speed was 60 fps. The difference between the signal-to-noise ratio of mKate2-K67R/R197H, TagRFP-T, and mKate2 localizations was assessed using the Kolmogorov–Smirnov test. Image acquisition and super-resolution reconstruction were performed using the NimOS 490 1.18.3.15066 software (ONI, Oxford, UK). Image reconstruction was done using default parameters. Data analysis was performed using the FiJi ImageJ 1.53f51 [29] and custom Python 3.9 scripts.

## RESULTS AND DISCUSSION

To clarify the roles played by particular amino acid substituents in the chromophore environment of FusionRed and mKate2 in the physicochemical distinctness of these fluorescent proteins (including their spectral differences and features of chromophore maturation), we conducted a systematic mutational analysis implying the introduction of single, double, and triple reciprocal substitutions (Fig. S1) at the key positions 67, 158, and 197, which had earlier been identified as ‘gatekeepers’ of the FusionRed chromophore behavior based on its crystal structure [20].

**Description of the reciprocal mutants**

Single mutations. Substitution at position 67 (Arg↔Lys) had differential effects on the parental proteins. Thus, the mKate2-K67R variant was found to have negligible absorption in the visible range and to be almost non-fluorescent; the mutation probably strongly affected folding and/or chromophore maturation. Conversely, FusionRed-R67K contains several well-marked spectral species, which likely correspond to different chromophore structures (Table 1, Fig. S2). Its absorption (being simultaneously fluorescence excitation) peaked at 389, 514, and 580 nm. The latter red emissive species (λ_abs/ex_ = 580 nm, λem = 610 nm) behaves similarly to the parental FusionRed, while both short-wave species are supposed to be the populations of immature chromophore.

We assumed that a blue-emitting spectral species (λ_abs/ex_ = 389 nm, λ_em_ = 450 nm) corresponds to the neutral GFP-type chromophore, which is the well-described intermediate of the DsRed chromophore maturation [30, 31]. A yellow fluorescent species (λ_abs/ex_ = 514 nm, λ_em_ = 522 nm) of FusionRed-R67K, which spectrally resembles conventional yellow fluorescent proteins (EYFP, TagYFP) that bear a GFP- chromophore π-stacked with the tyrosine-203 residue [32], is less usual for RFPs. As one can speculate, the R67K substitution led to partial "freezing" of the FusionRed chromophore maturation at the pre-last oxidation step (GFP-like chromophore), and an anionic GFP-chromophore (usually absorbing at 470–500 nm) underwent a bathochromic spectral shift (to the yellow species) due to its π-stacking with the imidazole ring of histidine-197.

**Table 1:** Summary of the spectral properties, chromophore maturation, and post-translational chemistry observed in the set of single, double, and triple reciprocal mutants of FusionRed and mKate2

Protein	Absorption peak, nm	λ_ex_/λ_em_, nm	EC^a^ (M^-1^·cm^-1^)	FQY^b^	Molecular brightness (EC · QY/1000)	Comment
FusionRed- R67K/C158A/H197R	376; 488; 583	376/409; 488/508; 583/616	n/d	< 0.05	n/d	poor maturation^*^
FusionRed- C158A/H197R	n/d	n/d	n/d	n/d	n/d	poor maturation^*^
FusionRed- R67K/H197R	380; 488; 584	380/449; 488/511; 584/616	n/d	0.62	n/d	
FusionRed- R67K/C158A	386; 513; 580	n/d	n/d	n/d	n/d	poor maturation^*^
FusionRed- H197R	n/d	570/607	n/d	< 0.01	n/d	poor maturation^*^
FusionRed-C158A	571	571/598	91 000	0.24	21.84	[20]
FusionRed-R67K	389; 514; 580	389/450; 514/522; 580/610	n/d	0.3	n/d	
FusionRed	580	580/608	94 500	0.19	17.9	[10]
mKate2	586	588/633	62 500	0.4	25	[21, 33]
mKate2-K67R	405; 588	n/d	n/d	n/d	n/d	poor maturation^*^
mKate2-A158C	380; 590	590/624	47 300	0.47	22.2	[20]
mKate2-R197H	385; 510; 582	510/520; 582/612	n/d	0.26	n/d	
mKate2- K67R/A158C	n/d	n/d	n/d	n/d	n/d	poor maturation^*^
mKate2- K67R/R197H	579	579/603	90	000	0.44	39.6
mKate2- A158C/R197H	380; 513; 583	380/435; 583/611	n/d	0.39	n/d	
mKate2- K67R/A158C/R197H	n/d	n/d	n/d	n/d	n/d	poor maturation^*^

Note: n/d – not determined.

^a^Molar extinction coefficient; EC has not been determined for the variants possessing several spectral species.

^b^Fluorescence quantum yield; FQY was measured for the red emissive species only.

^*^Label applied if a low-to-invisible bacterial biomass fluorescence 48 h post transformation and/or low relative absorbance at the chromophore-related wavelengths (e.g., A280/A580 > 10) was observed.

The influence of a reciprocal mutation at position 158 (Cys↔Ala) on the spectral properties of FusionRed and mKate2 has earlier been documented [20]. Although this substitution did not result in the formation of new spectral species or severe inhibition of the chromophore maturation (or strong changes in molecular brightness), we include the data on the corresponding mutants (FusionRed-C158A and mKate2- A158C) in Table 1 for uniformity.

The mutations at position 197 (His↔Arg) had effects that were antagonistic to those of R67K/K67R. Similarly to mKate2-K67R, FusionRed-H197R was found to possess negligible absorption in the visible spectral region and to be almost non-fluorescent due to hindrance in the chromophore maturation. mKate2-R197H has its the absorption maxima at 385, 510, and 582 nm, of which two latter are also the fluorescence excitation peaks, and emits at 520 nm and 612 nm (Table 1, Fig. S3). The identities and origins of these spectral species are suggested to be the same as those of FusionRed-R67K. Notably, there is a well-marked hypsochromic shift in the absorption/ emission maxima of the red species of mKate2-R197H compared to the parental mKate2 protein (582/612 nm vs. 588/633 nm), which proves the key role played by His-197 in the determination of the FusionRed spectral distinction.

Double mutations. The chromophore maturation in both proteins was generally less tolerant to the introduction of sets of two amino acid substitutions. Thus, three out of six double mutants were either extremely dim and weakly absorbing or almost non-fluorescent and having no detectable absorption maxima in the visible range (see Table 1). In the relatively bright FusionRed-R67K/H197R variant, an antagonistic functionality of the residues at positions 67 and 197 is expressed. The R67K mutation partially unlocks the chromophore maturation strongly inhibited by H197R. FusionRed-R67K/H197R possessed three emissive species (Table 1, Fig. S4): the blue-emitting neutral GFP (λ_abs/ex_ = 380 nm, λ_em_ = 449 nm), the green-emitting anionic GFP (λ_abs/ex_ = 488 nm, λ_em_ = 511 nm), and the red-emitting DsRed-like one (λ_abs/ex_ = 584 nm, λ_em_ = 616 nm). Remarkably, the red form of FusionRed-R67K/H197R showed a well-defined bathochromic shift in both absorption and emission (4 and 8 nm, respectively) compared to the parental FusionRed, thus providing additional evidence of the role of the substituent at position 197 in spectral tuning of RFPs. The mKate2-A158C/ R197H variant demonstrates a complex spectral behavior (Table 1, Fig. S5) similar to that observed for the FusionRed R67K and mKate2 R197H proteins. In mKate2-A158C/R197H, the R197H substitution is likely to provide a hypsochromic shift of the fluorescence spectra of the red species, as well as a stacking interaction with the immature "green" chromophore, leading to the formation of spectral species with an absorption maximum at 513 nm. Importantly, the latter was found to be non-fluorescent.

**Table 2:** Brief summary of the spectral properties possessed by mKate2, FusionRed, and mKate2-K67R/R197H, aka Diogenes

Protein	λ_ex_, nm	λ_em_, nm	EC (M^-1^·cm^-1^)	FQY	Molecular Brightness (EC · FQY/1000)	Fluorescence lifetime, ns^#^
mKate2	588	633	62,500	0.4	25	2.4^1^/2.05^1^
FusionRed	580	608	94,500	0.19	17.955	1.6^2^
mKate2-K67R/R197H	579	603	90,000	0.44	39.6	2.2^1^

^#^Intensity-weighted average lifetimes are shown. For mKate2, two lifetime values obtained at different setups are shown (see the “Fluorescence lifetime” section).

^1^Monoexponential fitting gave adequate goodness (χ^2^ ≤ 1.3);

^2^Biexponential fitting gave adequate goodness (χ^2^ ≤ 1.3).

**Fig. 1:**
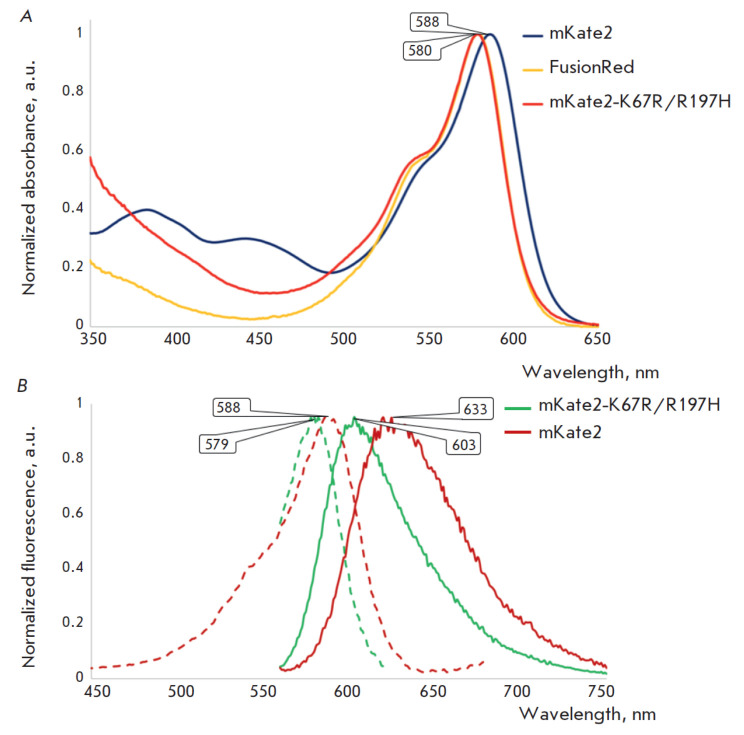
The absorption (*A*) and fluorescence (*B*) spectra of mKate2-K67R/R197H compared to those of its parent mKate2 and close homologue, FusionRed (absorption only). The wavelengths of the maxima of major bands are shown in the bubbles. In the fluorescence graph, dashed lines show fluorescence excitation; solid lines show fluorescence emission

mKate2-K67R/R197H was the only variant from the library of the mKate2/FusionRed reciprocal mutants that exhibited fast chromophore maturation and high molecular brightness. Its fluorescence quantum yield of 0.44 and extinction coefficient of 90,000 make it 1.6 times brighter than mKate2 and 2.2 times brighter than FusionRed protein. In contrast to the parental protein, this double mutant lacks the minor shortwave absorption peaks at ~ 390 and ~ 450 nm attributed to immature chromophore and exhibits a blue-shifted main absorption band with a pronounced ‘shoulder’ at ~ 540 nm, typical of FusionRed (Table 1, and Table 2, Fig. 1).

Triple mutations. The introduction of the full triad of reciprocal 67/158/197 substitutions had a striking effect on protein maturation. Thus, both triple mutants, mKate2-K67R/A158C/R197H and FusionRed-C158A/ H197R/R67K, displayed undetectable-to-very-low absorbance/fluorescence in the visible part of the spectrum (see Table 1), presumably indicating chromophore "freezing" in the early stages of maturation. The information value of these variants in terms of establishing the molecular determinants of the spectral distinctiveness of FusionRed/mKate2 turned out to be low.

Over all, our phenotypic analysis revealed that the substitutions at position 67 likely cause a shift of the chromophore within the beta-barrel, since, after the mutagenesis, the spectroscopic signs of its π-stacking interaction with histidine-197 (if any) are changed compared to those in the parental proteins. The impact of these substitutions on chromophore maturation is also evident: in all the cases, except for mKate2-K67R/R197H, they led to either a strong alteration in the maturation or at least an elevated presence of the shortwave spectral species representing maturation intermediates. Substitutions at position 197 induce spectral shifts, a bathochromic absorption/ emission shift in the case of the FusionRed-derived variants and a hypsochromic one in the mKate2 mutants. We speculate that the reason behind this phenomenon might have to do with the π-stacking interaction between the chromophore and histidine (occupying position 197 in the original FusionRed) switched off/on by reciprocal mutations. Additionally, these mutations were shown to have a noticeable impact on the chromophore maturation process.

Physicochemical properties of mKate2-K67R/R197H and its performance in microscopy Next, we endeavored to achieve a detailed characterization of the physicochemical properties of the mKate2-K67R/R197H protein, named Diogenes, by focusing on its performance in cellular fluorescence imaging.

Oligomeric state and protein labeling. The first step was to analyze the protein’s oligomeric state, which is among the key predictors of efficient low-disturbed/ minimally invasive labeling of intracellular targets. The gel filtration chromatography data (Figs. S6 and S7) show that the purified protein elutes as a single peak with an estimated molecular weight of ~38 kDa (at a concentration of up to at least 5 mg/mL). Since this molecular weight corresponds neither to the monomer (~25 kDa) nor to the dimer (~50 kDa), the gel filtration data cannot be interpreted unambiguously. One can assume that concentrated Diogenes in an aqueous solution is either a strict monomer or a strict dimer, having anomalous chromatographic mobility in either case. Alternatively, it is possible that we observed an equilibrium mixture of the monomeric and dimeric states.

It would be reasonable to expect that, in terms of their oligomeric state, Diogenes would be close to parental mKate2, which was originally described as a monomer [21], with further evidence of some propensity to oligomerize in aqueous solutions at a high concentration [10] and in cellulo [12]. Meanwhile, it is important to evaluate how the monomeric quality of this variant compares with that of its spectral analog, FusionRed. During the engineering of the latter, considerable effort was devoted to optimizing the outer surface of the beta-barrel, including the elimination of potentially dimerizing residues [10]. It was indeed shown that purified FusionRed behaves as a strict monomer [10] and scores higher on the monomericity ranking than mKate2 when examined in cellulo (91.5 ± 3.0% vs. 81.1 ± 6.1% in the OSER assay [12]). However, establishing a causal link between the monomerizing mutations introduced into FusionRed and its better performance in cellulo remains somewhat debatable, since the rational design of these substitutions was based on the spatial structure of mKate rather than on that of mKate2 [34]. Moreover, protein folding and the observed molecular interactions in crystals may not fully correspond to those in the aqueous phase [35, 36]. In any case, the ambiguous chromatographic picture for Diogenes prompted us to try to evaluate its oligomeric state in a cellular model system.

**Fig. 2:**
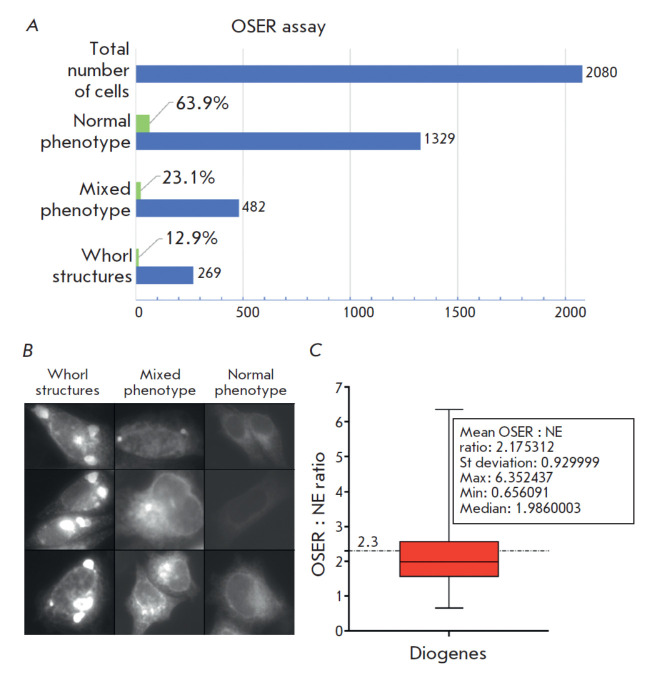
Summary of the Diogenes examination using the OSER assay. (*A*) Histogram showing the total cell number (HeLa) and distribution of three phenotypes between them; (*B*) The gallery of fluorescence images illustrating the phenotypes observed during the OSER-based examination in live HeLa cells. The "Whorl structures" column depicts the cells where homo-oligomerization of the label yielded the typical organized smooth endoplasmic reticulum (OSER) structures (whorls) as they have been earlier described [12, 23]. The "Mixed phenotype" row represents the cases when some labeling artifacts other than typical whorls (small puncta, dots, regions with locally increased brightness) are observed. The "Normal phenotype" row exemplifies the cells with an evenly stained ER tubular network; (*C*) Graph showing the quantitative analysis of the OSER : NE intensity ratio in live U2OS cells. The red box indicates 25–75 percentile of the readings; whiskers stand for the min and max read of the set. Median is illustrated by a horizontal line within the box; the dash-and-dot line at 2.3 indicates the monomer’s threshold OSER : NE value according to the original paper [23]. Descriptive statistics for the data are shown in the inset

**Fig. 3:**
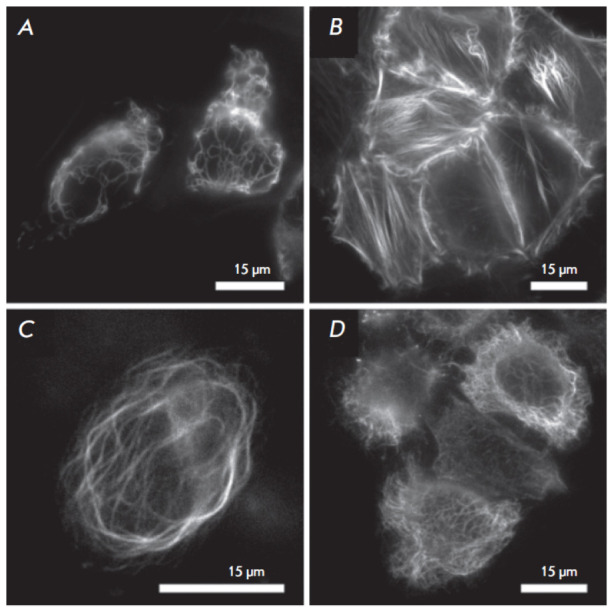
Fluorescent labeling of intracellular structures with Diogenes in live HeLa Kyoto cells. (*A*) Vimentin–Diogenes; (*B*) lifeact–Diogenes; (*C*) ensconsin–Diogenes; and (*D*) Diogenes–cytokeratin; Scale bars are 15 μm

To this end, we applied an OSER assay [23], which has become the de facto standard for assessing the monomerization of fluorescent proteins in cellulo [12, 37]. Since modern uses of the OSER assay often diverge from the original one, we performed the assessment in two different cell lines: HeLa for the widely-used simplified (OSER to non-OSER phenotypic) assessment and U2OS for the original (OSER : NE ratio) assessment. In HeLa, the analysis revealed ~ 87% whorl-free cells (Fig. 2A), which could be interpreted as a relatively high monomeric grade lying between the FusionRed and mKate2 scores published previously [12]. However, in addition to the obvious OSER-negative/positive cells, we observed a well-represented (~ 23%) cell fraction possessing diverse labeling features, such as small puncta, dots, local areas with increased brightness, which probably should not be attributed to a typical tubular ER phenotype (we labeled this population "mixed phenotype", see Fig. 2B for details). The aforementioned structures can be an indication of protein aggregation or its non-specific interaction with the intracellular environment, which could probably limit its efficiency in some circumstances. As per the original OSER analysis protocol in U2OS, the revealed mean OSER : NE ratio of Diogenes is 2.175, with the median value of 1.986 and standard deviation of 0.9299. Despite the relatively large standard deviation, both the mean OSER : NE ratio and the median allow us to consider Diogenes monomeric, with the monomericity borderline set at OSER : NE ≤ 2.3 ± 0.6 [23]. Finally, we assembled several mammalian expression constructs for visual evaluation of the effectiveness of mKate2-K67R/R197H when working in fusions. For this testing, we selected targets (cytoskeleton proteins) whose visualization quality, according to our experience, noticeably depended on the oligomeric status of the tag (Fig. 3). We subjectively rated the labeling quality as very high.

pH stability of Diogenes. Next, we compared the stability of fluorescence intensity between Diogenes and its relatives, mKate2 and FusionRed, within the wide pH range of 3–11 (Figs. S8 and S9). Over all, the protein exhibited a high pH stability, similar to that of mKate2, which is among the most pH-stable RFPs. Specifically, it could sustain a fluorescence level of ≥ 80% of the maximum within the most physiologically and biochemically relevant pH range of 6.5–9.5. In the acidic range (pH 3–6), Diogenes showed a lower relative brightness than FusionRed but slightly surpassed mKate2 (their pKa values were determined to be 6.1, 5.76 and 6.16, respectively). Unlike both counterparts, Diogenes fluorescence decreases abruptly in the strongly alkaline pH range of 10–11, although this acidity level is not that biologically relevant.

Fluorescence lifetime. We then measured the fluorescence decay kinetics of the purified mKate2-K67R/ R197H (Diogenes) in an aqueous solution using the time-correlated single photon counting approach and three different instrument setups (Figs. S10–S12). Importantly, the decay was shown to be monophasic in all the cases, with a lifetime value of ~ 2.2 ns. Surprisingly, in contrast to it and the FusionRed protein, which showed a biphasic fluorescence decay and a mean lifetime of ~ 1.6 ns with every setup used, the lifetime of the parental mKate2 was noticeably dependent on the measurement equipment. Thus, upon excitation with a 450 nm picosecond laser (FWHM ~100 ps, 20 MHz) and a 590 nm nanosecond pulsed LED (FWHM ~1.5 ns, 20 MHz), its lifetime was 2.4 ns (Figs. S10 and S12), while being only 2.05 ns upon excitation with a 590 nm femtosecond laser (FWHM ~150 fs, 80 MHz) (Fig. S11). The reasons for such flexibility remain unclear; they might be connected with some kind of excited-state processes known to occur in mKate2 and related proteins [30, 38]. Taking into account the excitation/emission wavelengths of Diogenes, mRuby [39] or mRuby2 [40] could be considered its close competitors in terms of fluorescence brightness/lifetime.

**Fig. 4:**
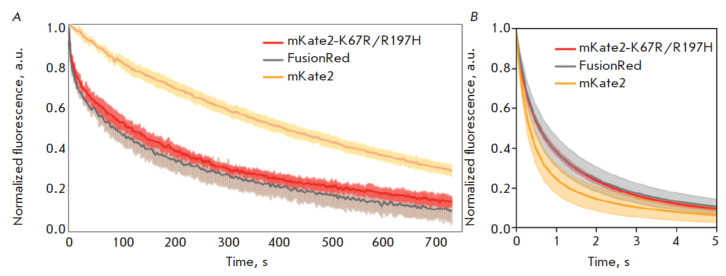
The photobleaching kinetics of the red fluorescent proteins mKate2-K67R/R197H (Diogenes), mKate2, and FusionRed measured in an aqueous solution of the purified protein at an excitation power density of ~ 2 W/cm2 (*A*) and live HeLa cells at ~ 1 kW/cm2 (*B*). Solid lines indicate the mean fluorescence intensity during photobleaching. Transparent areas indicate the standard deviation (five protein-containing particles or 20 cells for each fluorescent protein)

Photostability of Diogenes. High photostability is a desirable fluorophore property for both conventional fluorescence imaging and microscopy techniques with high spatial and temporal resolution [41]. Moreover, the photobleaching rate of a fluorescence protein may depend, non-linearly, on the excitation source power [12, 41]; this phenomenon can have a considerable impact when choosing a specific probe variant for a particular experiment. In this regard, we measured the photostability of Diogenes in two different model systems (Fig. 4). The photostability of purified mKate2-K67R/R197H measured in an aqueous environment (protein immobilized on microparticles) at a moderate power density typical of a widefield fluorescence microscope (~2 W/cm2) was found to be slightly higher than that of FusionRed (bleaching t1/2 215 s vs. 165 s) and significantly lower than that of mKate2 (t1/2 ~590 s, Fig. 4A). Surprisingly, in live HeLa cells upon high-intensity (~1 kW/cm2) excitation, typical of SMLM techniques, the new RFP showed better performance than mKate2 (ca. twofold higher photostability, Fig. 4B) and approximately the same as FusionRed, which is much dimmer and was, therefore, expected to be more photostable.

Single-molecule behavior of mKate2-K67R/R197H The increased photostability of Diogenes observed during imaging in a high excitation power mode, similar to that used in single-molecule microscopy techniques, prompted us to investigate the protein’s behavior at the single-molecule level. Preliminary runs performed using dSTORM-like settings of the super-resolution fluorescence microscope on droplets of the purified protein revealed a pronounced stochastic blinking behavior of Diogenes (data not shown). Critically, red and far-red FPs, including variants such as mScarlet, mKate2, TagRFP, FusionRed, and FusionRed-MQ, although exhibiting blinking behavior [28, 42, 43], mostly fell short of green fluorescent proteins in terms of single-molecule performance, with only a few exceptions [42]. Therefore, evaluating the potential of new RFP variants for various SMLM techniques, where spontaneous fluorescence blinking can be utilized to refine the localization of labeled molecules, is important.

**Fig. 5:**
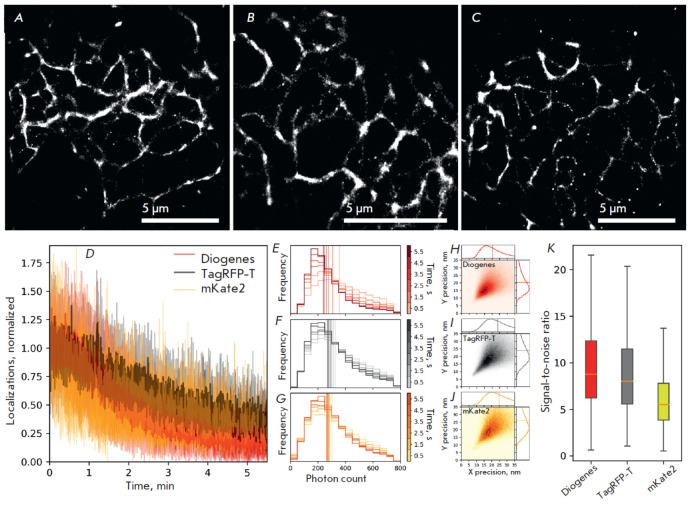
Comparison of the live-cell super-resolution imaging performance of mKate2-K67R/R197H, TagRFP-T, and mKate2 as parts of vimentin fusion proteins in live HeLa cells under the following imaging conditions: 2 kW/cm2 561 nm laser; 16.7 ms frame time; 20,000 frames. Super-resolution images of live HeLa cells transfected with vimentin–Diogenes, vimentin–TagRFP-T, and vimentin–mKate2, respectively (*A*), (*B*), (*C*); Scale bars are 5 μm. Stability of localization density of Diogenes, TagRFP-T, and mKate2 (*D*). The histogram of changes in the number of detected photons per single-molecule event over time of Diogenes, TagRFP-T, and mKate2, respectively; vertical lines represent the median values (*E*), (*F*), (*G*). 2D histograms of localization precision per single-molecule event of Diogenes, TagRFP-T, and mKate2, respectively (*H*), (*I*), (*J*); vertical lines on 1D histograms represent the median values. Signal-to-noise ratio of detected localizations (*K*); whiskers show the standard deviation, orange horizontal lines indicate median values

Here, we applied single-molecule localization microscopy (SMLM) to determine whether the Diogenes variant is capable of spontaneous blinking in cellulo and of visualizing intracellular structures with enhanced resolution. We chose the parental protein mKate2 and TagRFP-T, a protein known to have the strongest blinking pattern among the previously examined RFPs [42], as references. The single-molecule performance of Diogenes, TagRFP-T, and mKate2 was compared in a model system where these probes were fused with vimentin, transiently expressed in live HeLa cells, and monitored under conditions of super-resolution microscopy (Fig. 5).

Under the illumination of 2 kW/cm2 at 561 nm light and with a 16.7 ms frame time, all the proteins blinked at a single molecule level, allowing for the reconstruction of the sub-diffraction image of vimentin fibers in live HeLa cells (Fig. 5 A-C). Comparative analysis revealed that the stability of localization density was similar for all three proteins (Fig. 5D). Additionally, the difference in the median single-molecule brightness value was negligible among these proteins (Fig. 5 E-G). The localization accuracy of Diogenes appeared to be slightly higher than that of mKate2 and TagRFP-T (20.5 nm vs. 24 nm and 25 nm, respectively, Fig. 5 H-J), while the median value of the signal-to-noise ratio was ~1.5-fold higher for Diogenes than it was for mKate2, although the differences in the overall datasets for signal-to-noise values were insignificant (Fig. 5K). In conclusion, the performance of Diogenes in live-cell SMLM is on par or even slightly better than that of RFP, which was previously described as a promising probe for this microscopy modality [42]. However, it is still inferior in terms of the stability of localization density, molecular brightness, and localization precision to green fluorescent proteins capable of spontaneous blinking (e.g., mNeonGreen [44] and mBaoJin [45]). Intriguingly, additional 405 nm laser irradiation during imaging affected the density of localization of all three proteins (Fig. S13). Short violet laser flashes (illumination density of ~ 200 W/cm2) significantly increased the number of recorded localizations of all three proteins. Although this experiment is not sufficient to draw a conclusion about the nature of this phenomenon, it is possible that short-wave illumination may induce additional maturation of the chromophore and/or switch the chromophore from the long-lived dark state to the fluorescent state (e.g., via cis-trans isomerization of the chromophore).

## CONCLUSIONS

In this study, we systematically inspected the library of reciprocal mutants of the far-red fluorescent protein mKate2 and its daughter, the red FusionRed. We aimed to clarify the particular role of three residues in the chromophore environment (Arg/Lys-67, Cys/Ala-158, His/Arg-197) in determining the photophysical identities of these widely used genetically encoded probes.

One of the members of the constructed library, mKate2-K67R/R197H, named "Diogenes", exhibited a good combination of physicochemical and spectral properties, thus showing promise as a probe for conventional fluorescence microscopy techniques, as well as advanced imaging modalities of high spatial (SMLM) and temporal (FLIM) resolution. It inherits the advantages of both related proteins (FusionRed and mKate2). In particular, it possesses high fluorescence brightness, exhibits a monophasic fluorescence decay like mKate2, and shows good performance as a fusion tag similar to FusionRed. In terms of monomerization, Diogenes surpasses the parental mKate2 and possibly approaches the monomeric quality of FusionRed. The relatively high photostability of Diogenes (especially when normalized to the molecular brightness of the protein) under conditions of intense irradiation, as well as its remarkable ability for 405 nm illumination-induced photoactivation, which likely opens up possibilities for modulating its single-molecule behavior under live-cell multiphoton microscopy, are also worth noting. Our findings include indirect evidence that a smaller fraction of molecules trapped in long-lived transient dark states might be present in the population of Diogenes molecules (Fig. S10). Together with the absorption spectroscopy data (Fig. 1A), this may indicate a higher quality of chromophore maturation and steric adaptation inside the protein molecule compared to related RFPs.

It is important to note that, compared to its spectral analog, FusionRed, Diogenes carries a minimal number of mutations relative to the parental mKate2. Furthermore, the combination of substitutions (K67R/R197H) we found in the reciprocal library analysis was previously independently transferred from the bright but oligomerization-prone TagRFP protein to the dim monomer mKate2.5 to obtain FusionRed [10]. Like its relative FusionRed [18, 46], Diogenes can become a template for future semi-rational optimizations of RFPs, including those using high-throughput approaches.

## Abbreviations

FP: fluorescent protein
RFP: red fluorescent protein
FLIM: fluorescence lifetime imaging microscopy
BALM: bleaching/blinking assisted localization microscopy
OSER: organized smooth endoplasmic reticulum
NE: nuclear envelope
PEI: polyethyleneimine
SMLM: single molecule localization microscopy
